# Socioeconomic Status—Another Piece in the Puzzle of Access to Transplant

**DOI:** 10.1001/jamanetworkopen.2025.0580

**Published:** 2025-03-03

**Authors:** Daniel M. Guidot, Lisa M. McElroy

**Affiliations:** Geriatric Research, Education, and Clinical Center, Durham VA Medical Center, Durham, North Carolina; Department of Surgery, Duke University School of Medicine, Durham, North Carolina

Equitable access to transplant has emerged as a major field of study over the past several years.^1,2^ Transplant access is reduced for patients from marginalized groups, and a critical need is improved understanding about how patients progress from disease recognition to transplant candidacy determination. Chronic organ disease can remain silent for years, particularly among patients without reliable primary care. Patients can be referred for transplant from a variety of sources, including self-referral, primary care, and specialty care. Transplant centers vary in their processes for evaluating and selecting patients for transplant; lack of standardized criteria or reporting requirements makes the process vulnerable to multilevel biases. Over the past 5 years, there has been an influx of research attempting to overcome these challenges. National initiatives, including the release of a National Academies of Sciences, Engineering, and Medicine report^3^ and a recent Health Resources and Services Administration directive for modernization of the organ transplant system,^4^ have added opportunities for policies to eliminate structural root causes of inequitable access. Yet characterizing the drivers of disparities in access to transplant for marginalized groups remains a daunting task. The challenge reflects the multilevel complexities that ensue when patients with polymorbid chronic organ disease encounter large diverse teams and multistep conditional processes inherent to the transplant community.

Lehr et al^5^ took on these complexities in their analysis of patient-level data from the Cleveland Clinic Healthcare System linked to both the area deprivation index (ADI) and national US transplant registry data. They examined the association of socioeconomic position of patients with progression through the upstream phases of access to lung transplant and found that patients from the least-resourced ADI quintile were more likely to experience lapses of care early in the care continuum, less likely to reach the waiting list, and more likely to die across all care transitions. The authors found that a patient’s ADI was associated with different phases of care. A lower ADI was associated with a lower likelihood of being referred to pulmonary medicine but a higher likelihood of being referred for lung transplant. Similarly, patients with a low ADI who were referred for lung transplant were less likely to be waitlisted, but those listed experienced a similar rate of transplant as those in the most-resourced ADI. This suggests a higher severity of disease at the time of referral and perhaps a longer time to waitlisting after being referred for those with a low ADI. These trends held constant in their analysis of the access continuum by race and ethnicity. Non-Hispanic Black patients were more likely to transition to pulmonary medicine but were less likely to reach transplant evaluation. While similar trends were seen for waitlisting and transplant allocation for non-Hispanic Black patients, sample sizes were small, limiting the ability to achieve statistical significance. This study fits a critical gap in lung transplantation literature, highlighting a differential association with social deprivation based on phase of care preceding and during the transplant evaluation process.

It is likely that disparities in transplant, like other complex care, result from an intersection among biologic disease, social context, and care delivery. The analysis by Lehr et al^5^ contributes important new knowledge to this puzzle, but several questions remain worthy of further investigation. Lung transplant is indicated for 4 types of native lung disease: obstructive lung disease (eg, chronic obstructive pulmonary disease), pulmonary vascular disease (eg, pulmonary arterial hypertension), cystic fibrosis and immunodeficiency disorders, and restrictive lung disease (eg, interstitial lung disease). Each of these patient groups is subject to different disease trajectories and as such, likely requires distinct evaluation and approaches to improve access to transplant. This study focuses on obstructive and restrictive lung disease, and the study cohort reflects higher levels of obstructive lung disease represented in the earliest phases of care. While obstructive lung disease may make up the majority of adult lung disease, it is not the most common primary disease among lung transplant referees. As such, upstream findings may not generalize to the majority of patients who will progress to end-stage lung disease requiring transplantation. The study details very little of the referral pathways used by the Cleveland Clinic Healthcare System or the policies related to lung transplant eligibility; this is particularly important for the cohorts further along in the process that included out-of-state referrals for transplant evaluation. The ADI was also the sole measure of socioeconomic position, but the ADI as an area-based measure has important limitations for assessment of individual characteristics.^6^ The ADI also does not take into account several transplantspecific considerations such as distance to a transplant center or financial eligibility requirements.

Despite the influx of research and shifts in national policy, lung transplant is underrepresented in studies examining race and ethnicity or socioeconomic status–based disparities in access to transplant, especially in the prewaitlist period.^2^ By identifying the varying associations between the ADI and progression through the transplant continuum, Lehr et al^5^ help us better understand the complex association with socioeconomic position, individual demographics, and lung transplant access.
